# Supplementary material for: Polyfluorinated crosslinker-based solid polymer electrolytes for long-cycling 4.5 V lithium metal batteries
Source: Nat Commun. 2023 Apr 21;14:2301. doi: 10.1038/s41467-023-37997-6 (PMC10121557; doi:10.1038/s41467-023-37997-6)
Supplement: Supplementary file 1 — Supplementary Information [file 41467_2023_37997_MOESM1_ESM.pdf]

Supplementary Information

Polyfluorinated Crosslinker-based Solid Polymer Electrolytes for Long-Cycling 4.5 V Lithium Metal Batteries

Lingfei Tang[1,2†], Bowen Chen[1,2†], Zhonghan Zhang[3,4†], Changqi Ma[1,2†], Junchao Chen[5], Yage Huang[5], Fengrui Zhang[2], Qingyu Dong[2], Guoyong Xue[5], Daiqian Chen[2], Chenji Hu[5], Shuzhou Li[4], Zheng Liu[3,4], Yanbin Shen[1,2], Qi Chen[1,2]*, Liwei Chen[2,5,6]*

[1] School of Nano-Tech and Nano-Bionics, University of Science and Technology of China, Hefei 230026, China

[2] *i*-Lab, Suzhou Institute of Nano-Tech and Nano-Bionics, Chinese Academy of Sciences, Suzhou 215123, China

[3] CINTRA CNRS/NTU/THALES, UMI 3288, Research Techno Plaza, Singapore, 637553, Singapore

[4] School of Materials Science and Engineering, Nanyang Technological University, 50 Nanyang Ave, Singapore, 639798, Singapore

[5] School of Chemistry and Chemical Engineering, In situ Center for Physical Sciences, Shanghai Electrochemical Energy Device Research Center, and Frontiers Science Center for Transformative Molecules, Shanghai Jiao Tong University, Shanghai 200240, China

[6] Solid-State Battery Research Center, Global Institute of Future Technology, Shanghai Jiao Tong University, Shanghai 200240, China

Author Contributions

† These authors contributed equally to this work.

Corresponding authors

*E-mail: qchen2011@sinano.ac.cn; lwchen2018@sjtu.edu.cn

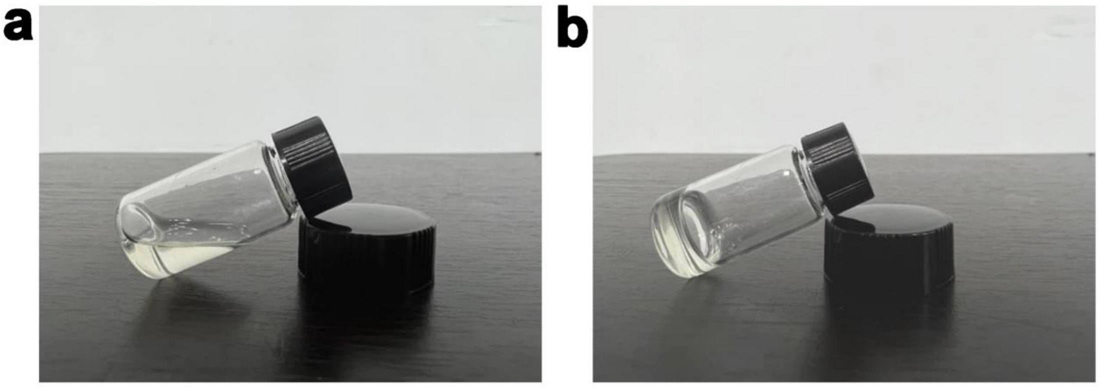

Supplementary Fig. 1 Photographs of SPE. Photographs of P(IL-OFHDODA-VEC) before (**a**) and after (**b**) polymerization.

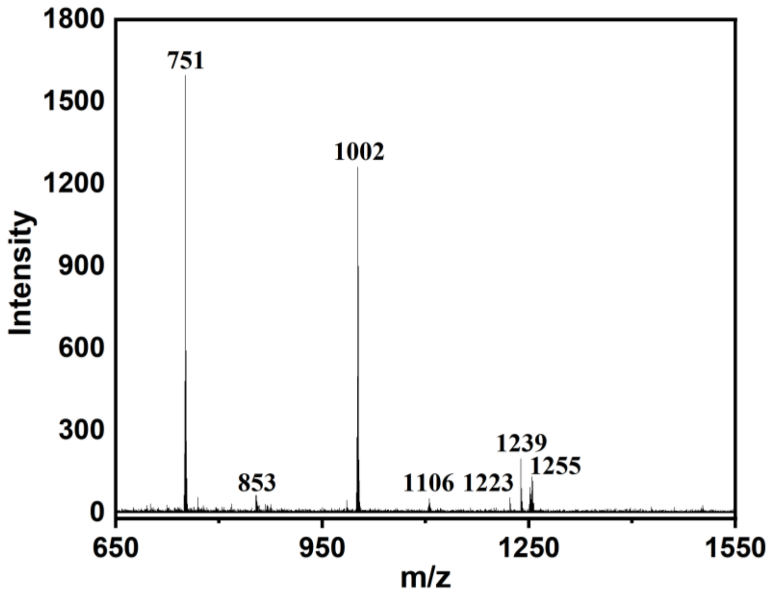

Supplementary Fig. 2 Mass spectrum of oligomers of the SPE extracted by diethyl ether.

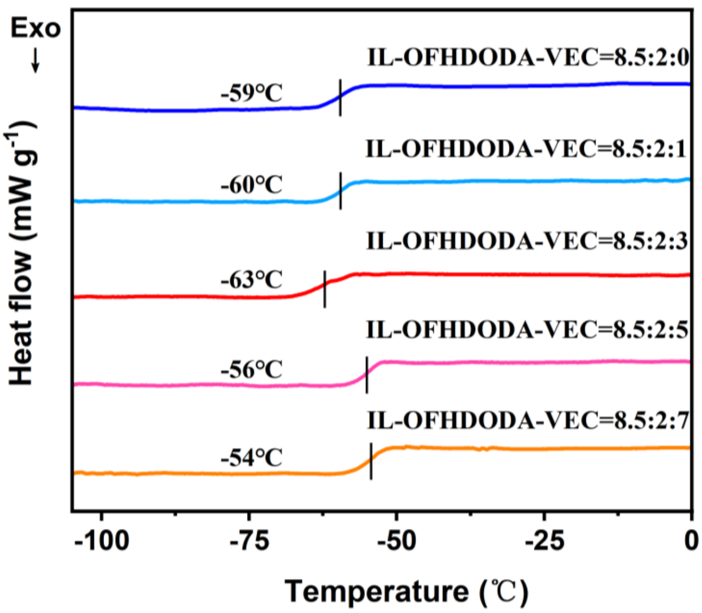

Supplementary Fig. 3 DSC curves of SPEs with different VEC ratio.

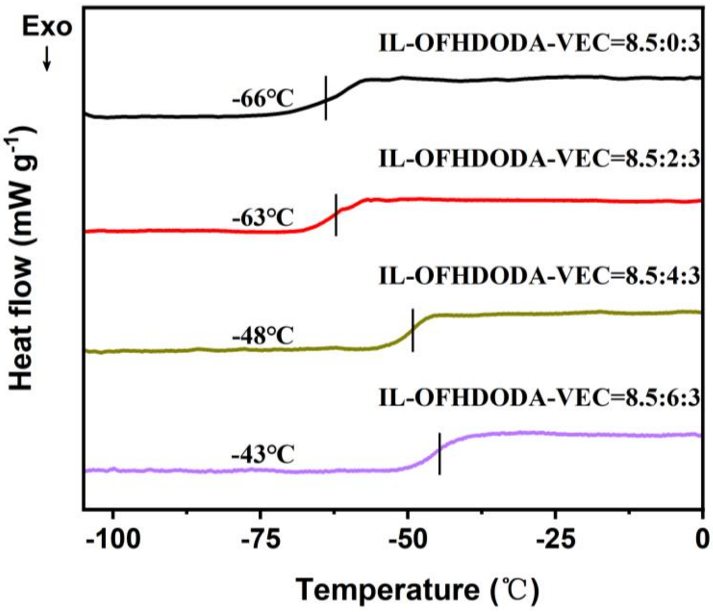

Supplementary Fig. 4 DSC curves of SPEs with different OFHDODA ratio.

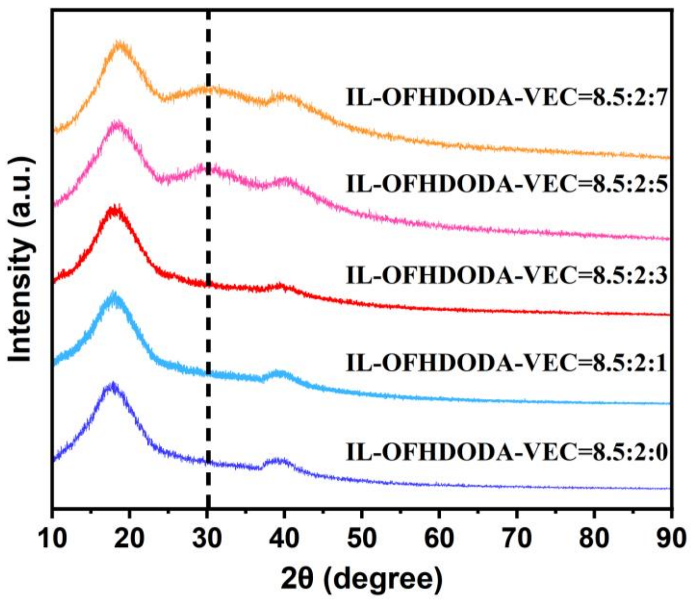

Supplementary Fig. 5 XRD patterns of SPEs with different VEC ratio.

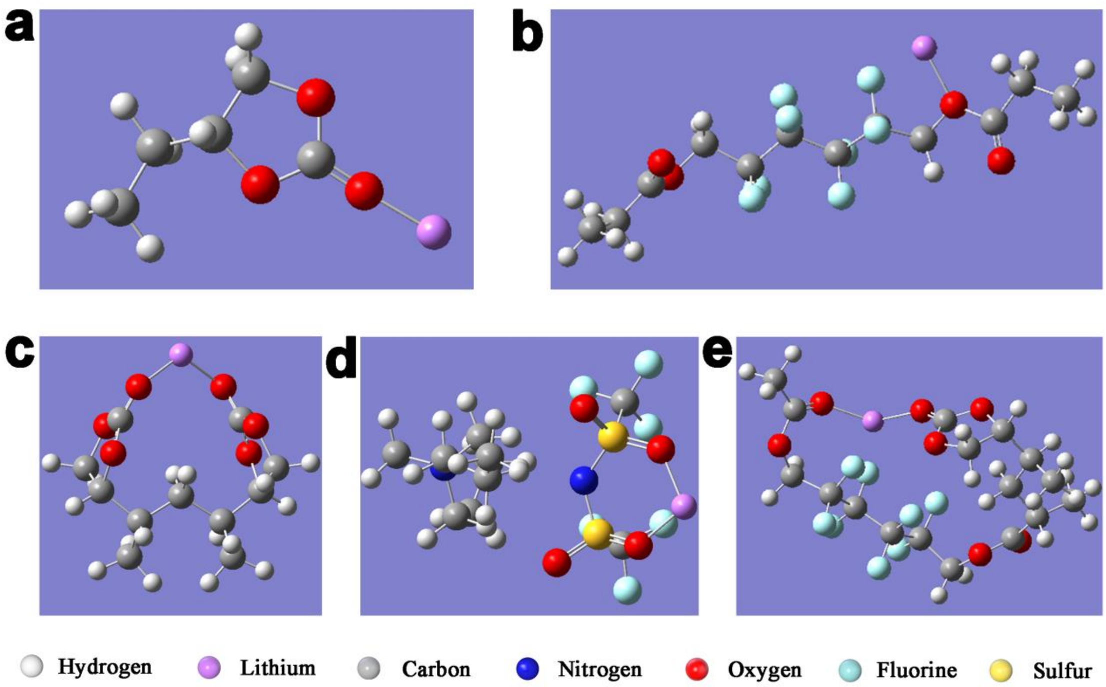

Supplementary Fig. 6 Optimized geometry of Li^+ absorbed near oxygen atoms of monomers or dimers of SPE via DFT calculation. **a** Li^+-O in the VEC monomer. **b** Li^+-O in the OFHDODA monomer. **c** O-Li^+-O in the VEC-VEC dimer. **d** O-Li^+-O in the IL monomer. **e** O-Li^+-O in the VEC-OFHDODA dimer.

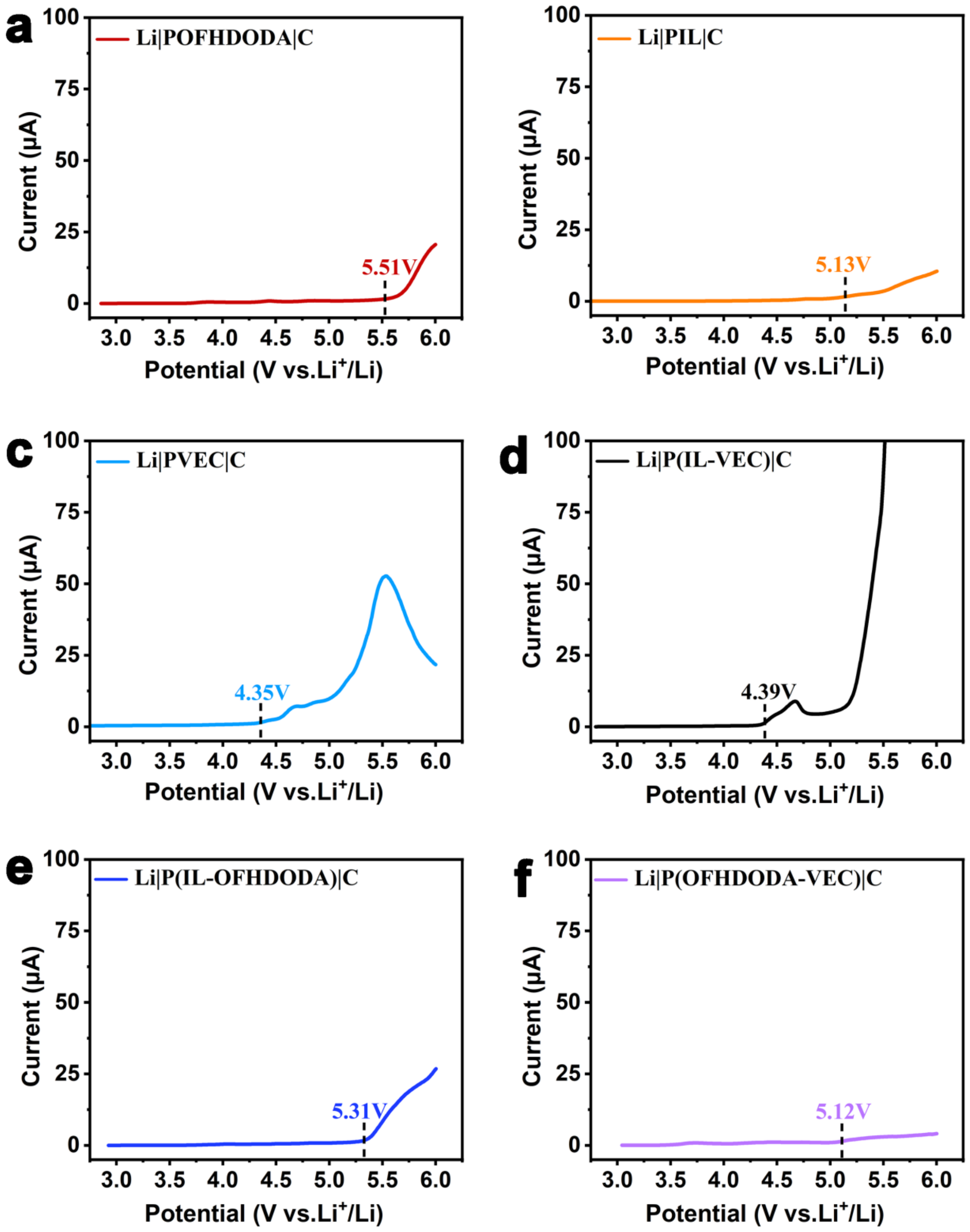

Supplementary Fig. 7 ESW of different components in SPE. LSV curves of POFHDODA (**a**), PIL (**b**), PVEC (**c**), P(IL-VEC) (**d**), P(IL-OFHDODA) (**e**) and P(OFHDODA-VEC) (**f**).

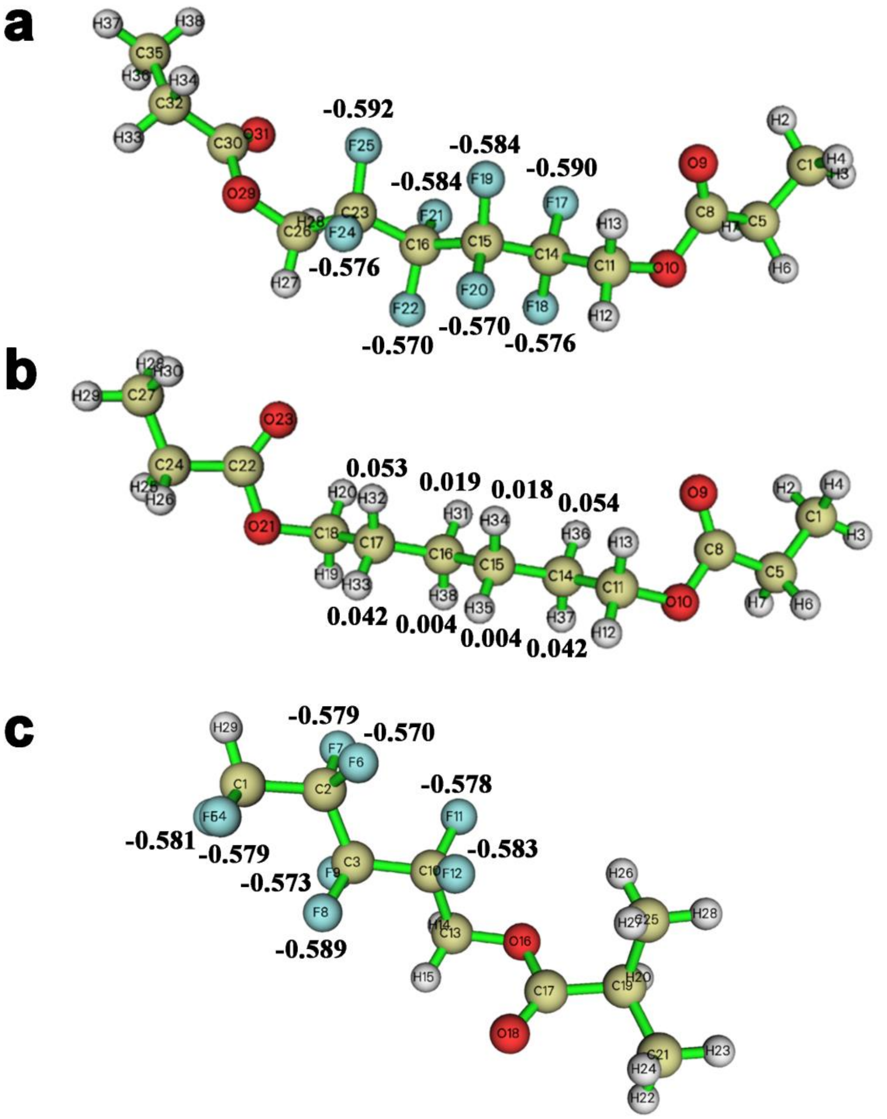

Supplementary Fig. 8 Partial charge of different monomers of SPE via DFT calculation. Partial charge of selected atoms of the OFHDODA (a), HDODA (b) and OFPMA (c).

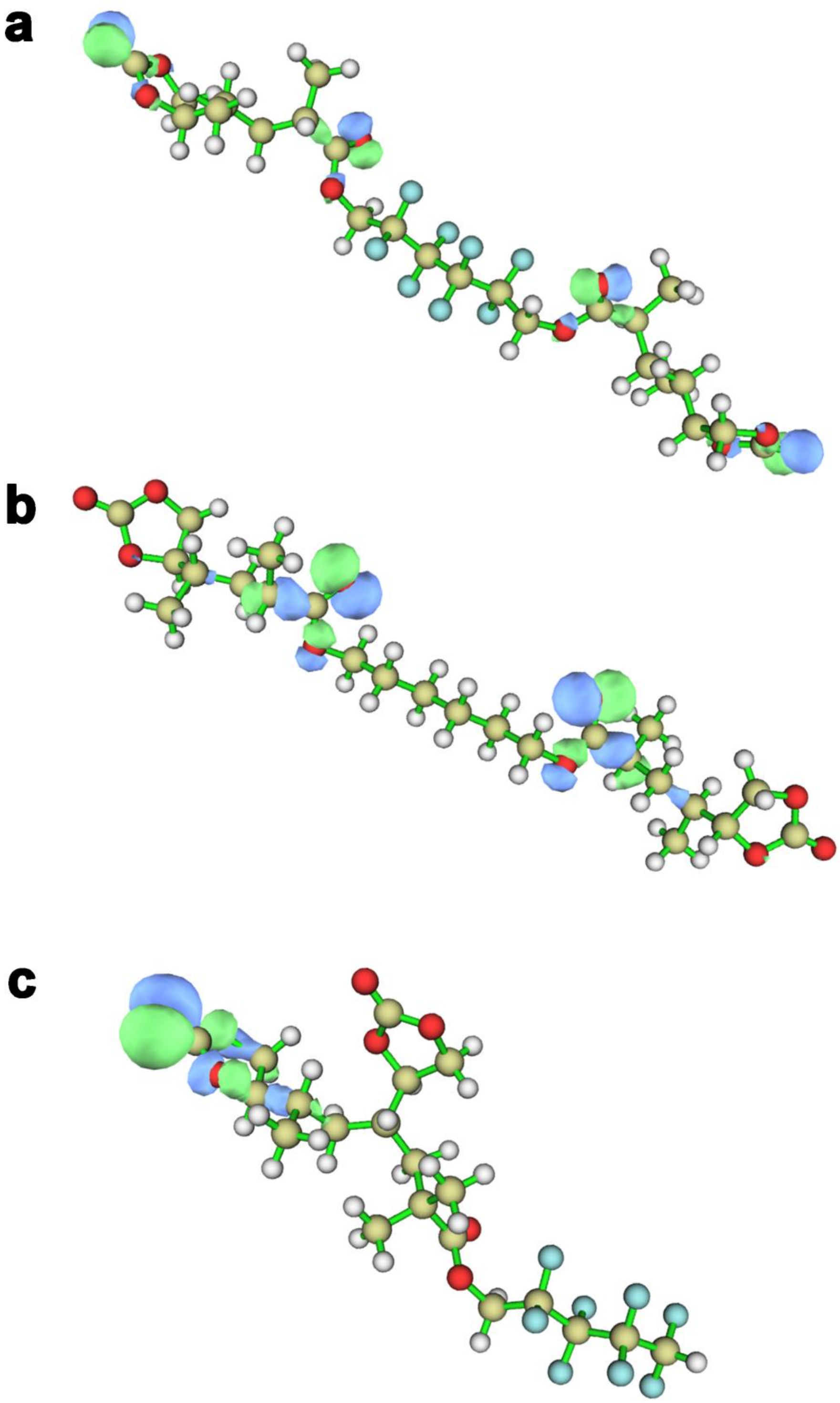

Supplementary Fig. 9 Frontier orbital information of optimized SPE trimers via DFT calculation. HOMO of the OFHDODA (a), HDODA (b) and OFPMA (c) copolymerized with two VEC.

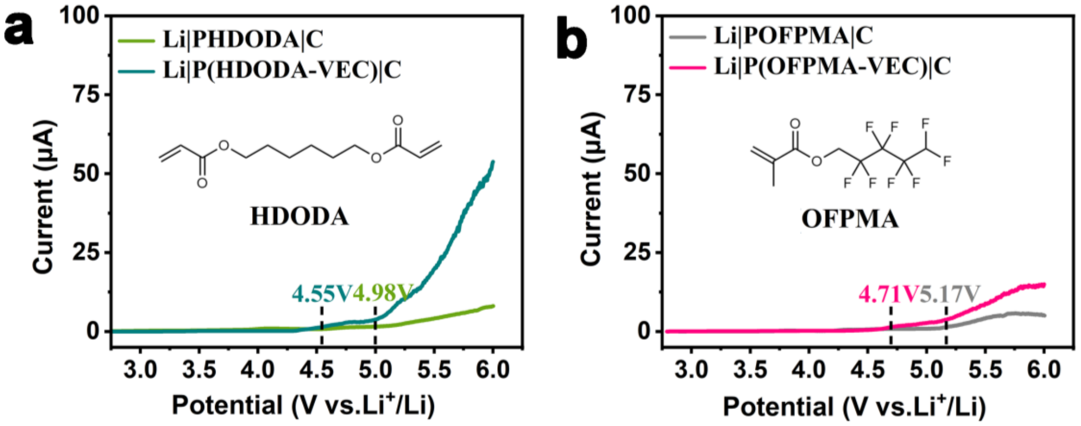

Supplementary Fig. 10 ESW of nonfluorinated crosslinkable and fluorinated non-crosslinkable SPEs with and without VEC. LSV curves of PHDODA and P(HDODA-VEC) (**a**), POFPMA and P(OFPMA-VEC) (**b**).

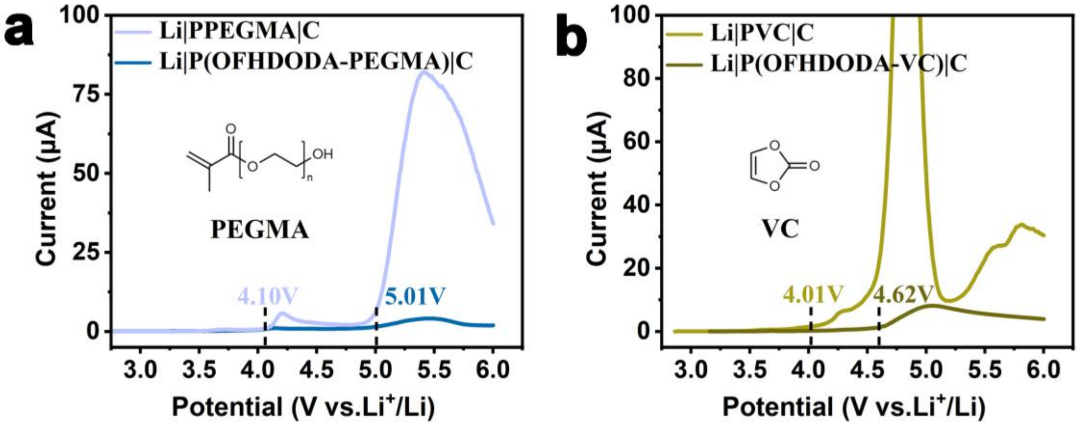

Supplementary Fig. 11 ESW of different SPEs containing oxygen-bearing polar groups with and without OFHDODA. LSV curves of PPEGMA and P(OFHDODA-PEGMA) (**a**), PVC and P(OFHDODA-VC) (**b**).

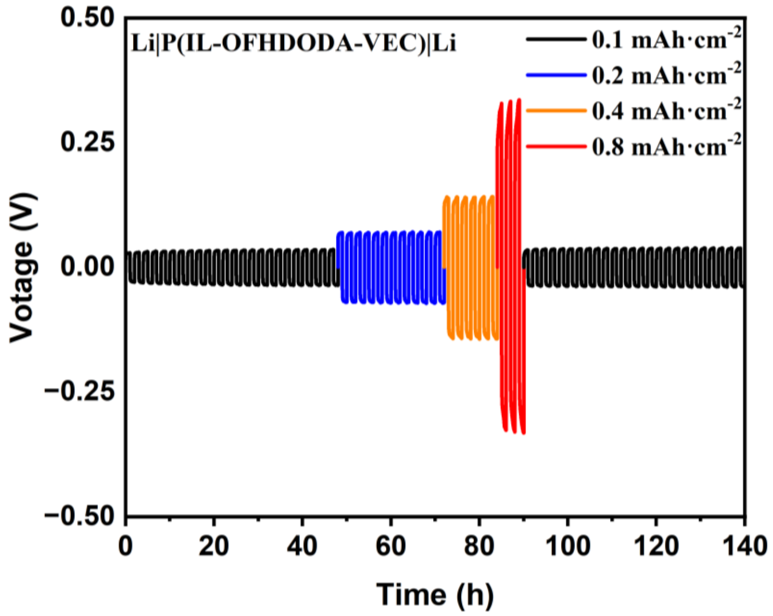

Supplementary Fig. 12 Galvanostatic cycling of the lithium symmetric cells with P(IL-OFHDODA-VEC) at current densities range from 0.1 to 0.8 mAh cm^{-2}.

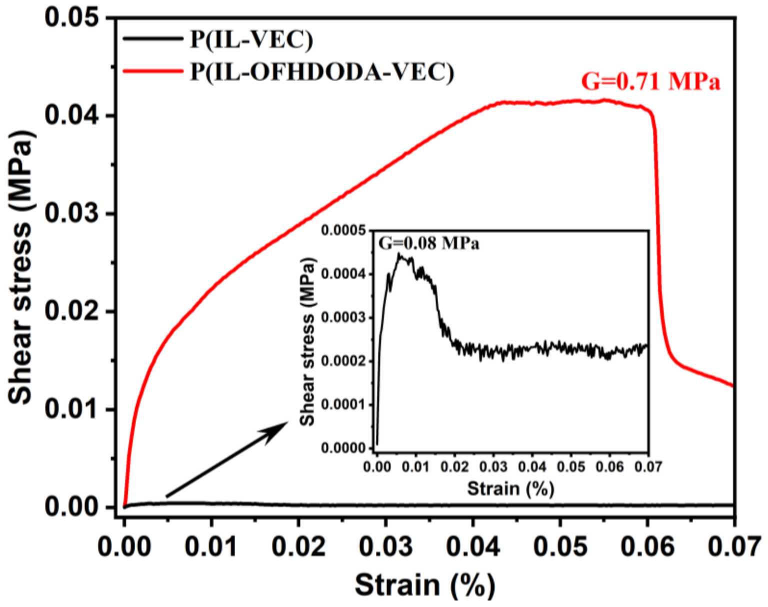

Supplementary Fig. 13 Shear modulus of P(IL-VEC) and P(IL-OFHDODA-VEC).

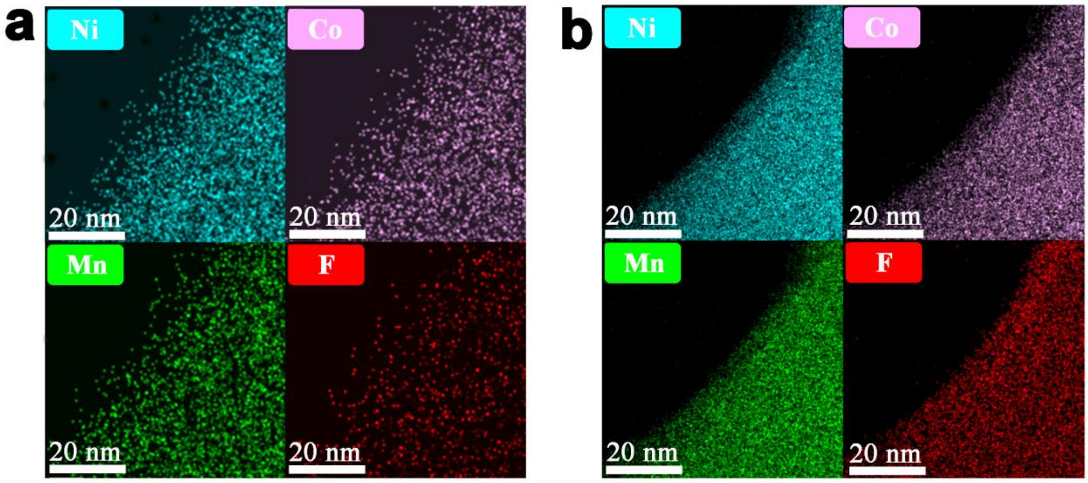

Supplementary Fig. 14 Elemental distribution in NCM523 cathode particles obtained from HRTEM-EDS. EDS mappings of Ni, Co, Mn and F elements of uncycled (a) and cycled (b) NCM523 cathode particles.

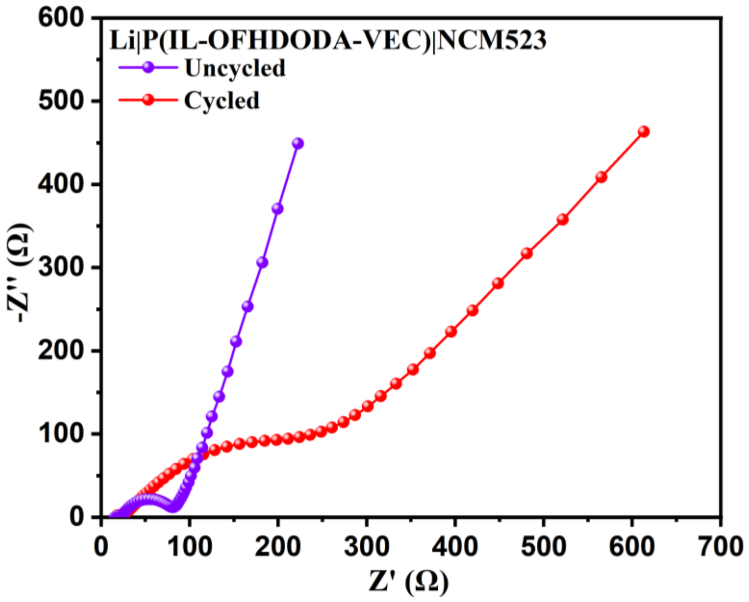

Supplementary Fig. 15 EIS of the Li|P(IL-OFHDODA-VEC)|NCM523 full cell before and after 200 cycles.

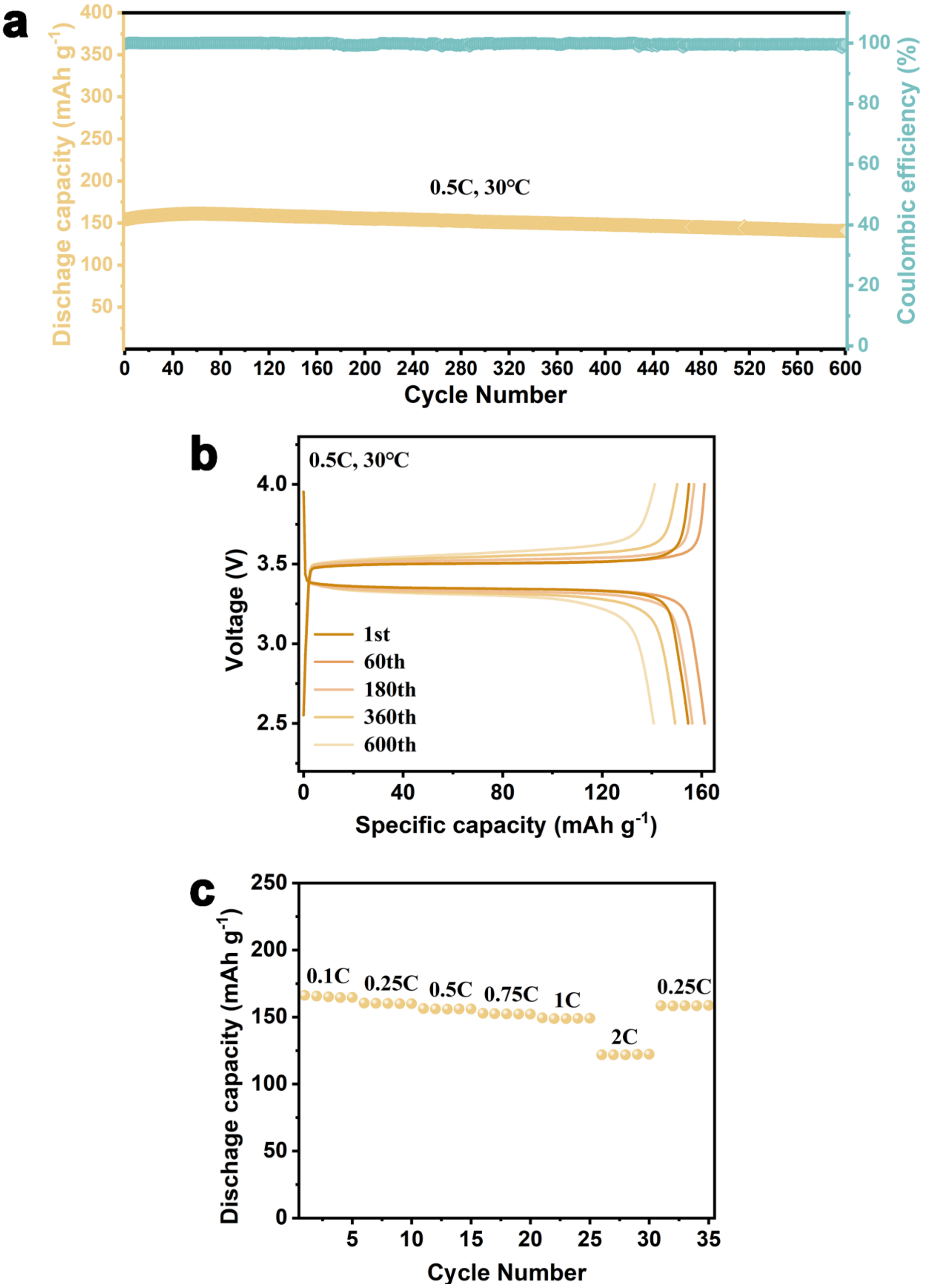

Supplementary Fig. 16 Electrochemical performance of the full cell with structure of Li|P(IL-OFHDODA-VEC)|LFP. a Cycling performance of Li|P(IL-OFHDODA-VEC)|LFP full cell at 0.5C. **b** 1-600th charge and discharge curves of Li|P(IL-OFHDODA-VEC)|LFP full cell. **c** Rate performance of Li|P(IL-OFHDODA-VEC)|LFP full cell.

Supplementary Table 1 Summary of τ and B at different VEC ratio

IL:OFHDODA:VEC	τ<br>μs	B<br>K
8.5:2:0	8.2	1014
8.5:2:1	6.4	934
8.5:2:3	1.7	825
8.5:2:5	5.0	1028
8.5:2:7	7.6	1040

Supplementary Table 2 Adsorption energy of Li^+ on O of different monomers and dimers of SPE via DFT calculation

Monomer or dimer	Adsorption type	Adsorption energy kJ mol^{-1}
VEC monomer	Li^+-O_{VEC}	-238.47
OFHDODA monomer	Li^+-$O_{OFHDODA}$	-262.46
VEC-VEC dimer	O_{VEC}-Li^+-O_{VEC}	-363.59
IL monomer	O_{IL}-Li^+-O_{IL}	-369.43
VEC-OFHDODA dimer	O_{VEC}-Li^+-$O_{OFHDODA}$	-418.02

Supplementary Table 3 Summary of a, conductivity, ESW, full cell performance at different crosslinking degree

IL:OFHDODA:VEC	a	Conductivity mS cm^{-1}	ESW V	t_{Li^+}	Initial discharge mAh g^{-1}	Capacity retention %	Rate retention %		
							0.1C	2C	2C/0.1C
8.5:0:3	/	1.73	4.39	0.13	96.96	45%@50	187.23	2.93	2%
8.5:2:3	0.90	1.37	5.08	0.40	162.92	96%@50	185.80	104.82	56%
8.5:4:3	0.85	0.13	5.36	0.48	148.97	90%@50	173.15	45.90	27%
8.5:6:3	0.79	0.03	5.46	0.49	123.61	85%@50	160.80	35.47	22%

Supplementary Table 4 Ionization energy of the OFHDODA, HDODA and OFPMA copolymerized with two VEC

Trimer	Neutral state energy<br>eV	Cation state energy<br>eV	Ionization energy<br>eV
VEC-OFHDODA-VEC	-67605.44	-67596.24	9.19
VEC-HDODA-VEC	-45996.44	-45987.47	8.97
VEC-OFPMA-VEC	-59262.13	-59253.22	8.91

Supplementary Table 5 Summary of τ and B at different OFHDODA ratio

IL:OFHDODA:VEC	τ<br>μs	B<br>K
8.5:0:3	/	/
8.5:2:3	1.7	825
8.5:4:3	10.9	1298
8.5:6:3	75.6	1540

Supplementary Table 6 Bulk Young's modulus of P(IL-VEC) and P(IL-OFHDODA-VEC)

Sample	Freq.<br>Hz	Young's modulus<br>MPa
P(IL-VEC)	45	/
P(IL-OFHDODA-VEC)	45	115±34

Supplementary Table 7 Summary of state-of-the-art Li|SPE|NCM full cell performance

SPE based LMBs	Conductivity of SPE<br>mS cm^{-1}	Rate<br>C	Initial discharge<br>mAh g^{-1}	Cutoff voltage<br>V	Cycle	Capacity retention<br>%	Ref.
Li|P(IL-OFHDODA-VEC)|NCM523	1.37@25℃	0.5	164	4.5	200@30℃	90	<u>This work</u>
Li|s-CPEG|NCM523	0.18@30℃	0.1	135	4.2	17@60℃	92	1
Li|PT-PEO-PT|NCM523	0.11@40℃	0.2	111	4.2	75@40℃	80	2
Li|FB|NCM523	0.15@70℃	0.1	124	4.2	10@70℃	97	3
Li|PEM|NCM523	1.99@30℃	0.5	142	4.3	50@40℃	89	4
Li|PEGDME-4|NCM523	0.15@60℃	0.2	165	4.5	200@N/A	59	5
Li|PDADMA FSI|NCM111	0.07@80℃	0.05	188	4.3	50@80℃	68	6
Li|poly-DOL|NCM622	1.10@25℃	0.1	160	4.2	5@N/A	96	7
Li|DLPE|NCM622	0.01-0.05@70℃	0.05	160	4.2	80@70℃	80	8
Li|XPEEC-1|NCM622	0.02@25℃	0.1	141	4.3	100@25℃	90	9
Li|P-P(20)|NCM811	0.45@60℃	0.1	162	4.2	50@60℃	75	10
Li|FMC-ASPE-Li|NCM811	0.64@80℃	0.3	112	4.2	100@70℃	87	11
Li|FPCSPE3-40|NCM811	0.05@25℃	0.1	218	4.5	200/300@25℃	85/70	12

Supplementary Note 1: Dielectric spectra analysis

To reveal the change in crosslinking degree of the SPE with different polyfluorinated crosslinker OFHDODA concentration, the dielectric spectra of segmental process at high frequencies has been analyzed based on the Havriliak – Negami (HN) function after removing the contribution of ohmic conduction as expressed in Eq. 1[13]:

$$\varepsilon^{*}_{\mathrm{NH}}(\omega) = \varepsilon_{\infty} + \frac{\Delta\varepsilon}{(1+(i\omega\tau)^{\mathrm{a}})^{\mathrm{b}}} \tag{1}$$

where $\varepsilon^{*}_{\mathrm{NH}}(\omega)$ is the complex dielectric function, ε_{∞} is the high frequency dielectric constant, Δε is the dielectric intensity, τ is the relaxation time of segment movement, a and b are the fractional shape parameters that describe the symmetric and asymmetric broadening of the complex dielectric function when a> 0, ab≤ 1 holds. The a is inversely correlated with the crosslinking degree.[14] The τ obtained by a least square method fitting the HN-function[15] (Eq.1) refers to the time required to transition from an equilibrium state to a new equilibrium state under the action of an external field, which is inversely correlated with the chain segment movement capability. The apparent activation energy (B) of the segment movement, that is also inversely correlated with the chain segment movement capability, can be extracted from the temperature dependent τ from -10 to -40 °C based on Vogel-Fulcher-Tammann (VFT) equation as expressed in Eq. 2:

$$\tau = \tau_0 e^{\frac{-\mathrm{B}}{(T-\mathrm{T}_0)}} \tag{2}$$

where τ_0 is the pre-exponential factor, T is the temperature, T_0 ($T_0 = T_g$ - 50K) is the ideal glass transition temperature.

Supplementary References

1. Zhao, Y.*, et al.* Polymer electrolyte with dual functional groups designed via theoretical calculation for all-solid-state lithium batteries. *J. Power Sources* **450**, 227614 (2020).
2. Zheng, J.*, et al.* Double Ionic-Electronic Transfer Interface Layers for All-Solid-State Lithium Batteries. *Angew. Chem. Int. Ed.* **60**, 18448-18453 (2021).
3. Jia, M.*, et al.* Fluorinated Bifunctional Solid Polymer Electrolyte Synthesized under Visible Light for Stable Lithium Deposition and Dendrite-Free All-Solid-State Batteries. *Adv. Funct. Mater.* **31**, 2101736 (2021).
4. Liu, F.*, et al.* Polymer Electrolyte Membrane with High Ionic Conductivity and Enhanced Interfacial Stability for Lithium Metal Battery. *ACS Appl. Mater. Interfaces* **12**, 22710-22720 (2020).
5. Yang, X.*, et al.* Determining the limiting factor of the electrochemical stability window for PEO-based solid polymer electrolytes: main chain or terminal –OH group? *Energy Environ. Sci.* **13**, 1318-1325 (2020).
6. Wang, X.*, et al.* Poly(Ionic Liquid)s-in-Salt Electrolytes with Co-coordination-Assisted Lithium-Ion Transport for Safe Batteries. *Joule* **3**, 2687-2702 (2019).
7. Zhao, Q., Liu, X., Stalin, S., Khan, K. & Archer, L. A. Solid-state polymer electrolytes with in-built fast interfacial transport for secondary lithium batteries. *Nat. Energy* **4**, 365-373 (2019).
8. Arrese-Igor, M.*, et al.* Toward High-Voltage Solid-State Li-Metal Batteries with Double-Layer Polymer Electrolytes. *ACS Energy Lett.* **7**, 1473-1480 (2022).
9. Jung, Y. C., Park, M. S., Kim, D. H., Ue, M., Eftekhari, A. & Kim, D. W. Room-Temperature Performance of Poly(Ethylene Ether Carbonate)-Based Solid Polymer Electrolytes for All-Solid-State Lithium Batteries. *Sci. Rep.* **7**, 17482 (2017).
10. Yang, L.*, et al.* The plasticizer-free composite block copolymer electrolytes for ultralong lifespan all-solid-state lithium-metal batteries. *Nano Energy* **100**, 107499 (2022).
11. Su, Y.*, et al.* Rational design of a topological polymeric solid electrolyte for high-performance all-solid-state alkali metal batteries. *Nat. Commun.* **13**, 4181 (2022).
12. Wang, Y., Chen, S., Li, Z., Peng, C., Li, Y. & Feng, W. In-situ generation of fluorinated polycarbonate copolymer solid electrolytes for high-voltage Li-metal batteries. *Energy Storage Mater.* **45**, 474-483 (2022).
13. Wübbenhorst, M. & Turnhout, J. V. Analysis of complex dielectric spectra. I. One-dimensional derivative techniques and three-dimensional modelling. *J. Non-Cryst. Solids* **305**, 40–49 (2002).
14. Glatz-Reichenbach, J. K. W., Sorriero, L. J. & Fitzgerald, J. J. Influence of Cross-Linking on the Molecular Relaxation of an Amorphous Copolymer Near Its Glass-Transition Temperature. *Macromolecules* **27**, 1338-1343 (1994).

15. Huang Y., Mei X. & Guo Y. Segmental and interfacial dynamics quantitatively determine ion transport in solid polymer composite electrolytes. *J. Appl. Polym. Sci.* **139**, 52143 (2021).
