# Supplementary material for: Polyfluorinated crosslinker-based solid polymer electrolytes for long-cycling 4.5 V lithium metal batteries
Source: Nat Commun. 2023 Apr 21;14:2301. doi: 10.1038/s41467-023-37997-6 (PMC10121557; doi:10.1038/s41467-023-37997-6)
Supplement: Supplementary file 3 — Description of Additional Supplementary Files [file 41467_2023_37997_MOESM3_ESM.pdf]

Description of Additional Supplementary Files

File Name: Supplementary Movie 1
Description: Flammability of P(IL-OFHDODAVEC)
